# (*E*)-*N*′-[4-(Dimethyl­amino)­benzyl­idene]-4-hy­droxy­benzohydrazide hemihydrate

**DOI:** 10.1107/S1600536810020763

**Published:** 2010-06-05

**Authors:** Huanyu Liu

**Affiliations:** a School of Chemistry and Chemical Engineering, Guangdong Pharmaceutical University, Zhongshan 528453, People’s Republic of China

## Abstract

In the title compound, C_16_H_17_N_3_O_2_·0.5H_2_O, the two hydrazide mol­ecules are approximately planar: the dihedral angles between the two substituted benzene rings are 7.7 (2) and 4.2 (2)°. Both hydrazone mol­ecules exist in a *trans* geometry with respect to their methyl­idene units. In the crystal, the water mol­ecule lies between the two organic mol­ecules and makes bifurcated O—H⋯(N,O) hydrogen bonds to both of them. The hydrazide mol­ecules form N—H⋯O and O—H⋯O hydrogen bonds, resulting in a three-dimensional network.

## Related literature

For the biological activity of hydrazone compounds, see: Banerjee *et al.* (2009 ▶). For the structures of hydrazone compounds, see: Ahmad *et al.* (2010 ▶); Li *et al.* (2010 ▶); Naveenkumar *et al.* (2010 ▶); Zhang (2009 ▶); Fun *et al.* (2008 ▶).
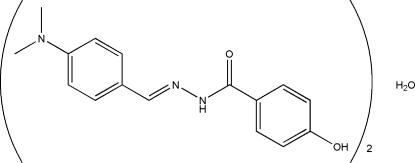


## Experimental

### 

#### Crystal data

C_16_H_17_N_3_O_2_·0.5H_2_O
*M* *_r_* = 292.34
Monoclinic, 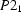

*a* = 6.1514 (9) Å
*b* = 18.098 (3) Å
*c* = 13.356 (2) Å
β = 95.489 (2)°
*V* = 1480.1 (4) Å^3^
*Z* = 4
Mo *K*α radiation
μ = 0.09 mm^−1^
*T* = 298 K
0.28 × 0.27 × 0.27 mm

#### Data collection

Bruker SMART CCD diffractometer
Absorption correction: multi-scan (*SADABS*; Sheldrick, 1996 ▶) *T*_min_ = 0.975, *T*_max_ = 0.976
8336 measured reflections
3254 independent reflections
2593 reflections with *I* > 2σ(*I*)
*R*_int_ = 0.028

#### Refinement

*R*[*F* ^2^ > 2σ(*F* ^2^)] = 0.037
*wR*(*F* ^2^) = 0.094
*S* = 1.04
3254 reflections
407 parameters
6 restraints
H atoms treated by a mixture of independent and constrained refinement
Δρ_max_ = 0.13 e Å^−3^
Δρ_min_ = −0.11 e Å^−3^

### 

Data collection: *SMART* (Bruker, 1998 ▶); cell refinement: *SAINT* (Bruker, 1998 ▶); data reduction: *SAINT*; program(s) used to solve structure: *SHELXS97* (Sheldrick, 2008 ▶); program(s) used to refine structure: *SHELXL97* (Sheldrick, 2008 ▶); molecular graphics: *SHELXTL* (Sheldrick, 2008 ▶); software used to prepare material for publication: *SHELXTL*.

## Supplementary Material

Crystal structure: contains datablocks global, I. DOI: 10.1107/S1600536810020763/hb5475sup1.cif

Structure factors: contains datablocks I. DOI: 10.1107/S1600536810020763/hb5475Isup2.hkl

Additional supplementary materials:  crystallographic information; 3D view; checkCIF report

## Figures and Tables

**Table 1:** Hydrogen-bond geometry (Å, °)

*D*—H⋯*A*	*D*—H	H⋯*A*	*D*⋯*A*	*D*—H⋯*A*
N5—H5*C*⋯O2^i^	0.90 (1)	2.56 (3)	3.171 (3)	125 (3)
N2—H2*A*⋯O5^ii^	0.90 (1)	2.12 (1)	3.003 (3)	168 (3)
O4—H4⋯O1^iii^	0.82	1.90	2.688 (2)	160
O2—H2*B*⋯O3^iv^	0.82	1.89	2.693 (3)	166
O5—H5*A*⋯O3	0.85 (1)	2.43 (2)	3.178 (3)	146 (3)
O5—H5*A*⋯N4	0.85 (1)	2.34 (2)	3.038 (3)	140 (3)
O5—H5*B*⋯O1	0.85 (1)	2.36 (2)	3.100 (3)	145 (3)
O5—H5*B*⋯N1	0.85 (1)	2.61 (2)	3.375 (3)	150 (3)

Symmetry codes: (i) 
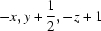
; (ii) 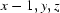; (iii) 
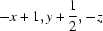
; (iv) 
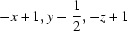
.

**Fig. 1.**
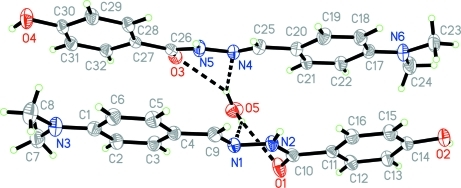
Molecular structure of (I) with 30% probability displacement ellipsoids. Hydrogen bonds are shown as dashed lines.

**Fig. 2.**
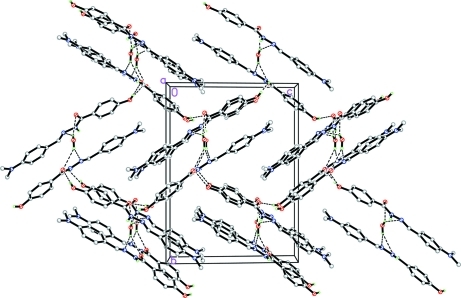
Packing structure of (I), viewed along the a axis. Hydrogen bonds are shown as dashed lines.

## supplementary crystallographic information

### Comment

In recent years, much attention has been focused on the biological properties of hydrazone compounds (e.g. Banerjee *et al.*, 2009). A number of hydrazone compounds have been prepared and investigated for their structures (Ahmad *et al.*, 2010; Li *et al.*, 2010; Naveenkumar *et al.*, 2010; Zhang, 2009; Fun *et al.*, 2008). In the present work, a new hydrazone compound with interesting structure is reported.

The title compound consists of two hydrazone molecules and one water molecule (Fig. 1). The two hydrazone molecules are approximately parallel to each other, and are linked together by the water molecule through O—H···N and O—H···O hydrogen bonds (Table 1). Both hydrazone molecules exist in *trans* geometry with respect to the methylidene units. The dihedral angles between the two substituted benzene rings in the hydrazone molecules are 7.7 (2) and 4.2 (2)°.

In the crystal structure, the hydrazone molecules and the water molecules are linked through N—H···O, O—H···O and O—H···N hydrogen bonds (Table 1), forming a three dimensional network (Fig. 2).

### Experimental

4-Dimethylaminobenzaldehyde (1.0 mmol, 149 mg) and 4-hydroxybenzohydrazide (1.0 mmol, 152 mg) were mixed in 50 ml methanol. The mixture was stirred at ambient temperature for 2 h and filtered. Colorless blocks of (I) were formed by slow evaporation of the filtrate for a week; presumably water was incorporated from the atmosphere.

### Refinement

The amino hydrogen atoms were located in a difference map and refined isotropically, with the N—H distance restrained to 0.90 (1)Å. Other hydrogen atoms were placed in calculated positions (C—H = 0.93 - 0.96 Å, O—H = 0.82 Å) and refined as riding with U_iso_(H) = 1.2U_eq_(C) and 1.5U_eq_(O and C_methyl_). In the absence of significant anomalous scattering effects, Friedel pairs were averaged.

### Crystal data

**Table:**

C_16_H_17_N_3_O_2_·0.5H_2_O	*F*(000) = 620
*M**_r_* = 292.34	*D*_x_ = 1.312 Mg m^−^^3^
Monoclinic, *P*2_1_	Mo *K*α radiation, λ = 0.71073 Å
Hall symbol: P 2yb	Cell parameters from 2665 reflections
*a* = 6.1514 (9) Å	θ = 2.6–24.5°
*b* = 18.098 (3) Å	µ = 0.09 mm^−^^1^
*c* = 13.356 (2) Å	*T* = 298 K
β = 95.489 (2)°	Block, colorless
*V* = 1480.1 (4) Å^3^	0.28 × 0.27 × 0.27 mm
*Z* = 4	

### Data collection

**Table:**

Bruker SMART CCD diffractometer	3254 independent reflections
Radiation source: fine-focus sealed tube	2593 reflections with *I* > 2σ(*I*)
graphite	*R*_int_ = 0.028
ω scans	θ_max_ = 27.0°, θ_min_ = 2.3°
Absorption correction: multi-scan (*SADABS*; Sheldrick, 1996)	*h* = −7→6
*T*_min_ = 0.975, *T*_max_ = 0.976	*k* = −23→23
8336 measured reflections	*l* = −9→17

### Refinement

**Table:**

Refinement on *F*^2^	Secondary atom site location: difference Fourier map
Least-squares matrix: full	Hydrogen site location: inferred from neighbouring sites
*R*[*F*^2^ > 2σ(*F*^2^)] = 0.037	H atoms treated by a mixture of independent and constrained refinement
*wR*(*F*^2^) = 0.094	*w* = 1/[σ^2^(*F*_o_^2^) + (0.0535*P*)^2^ + 0.0137*P*] where *P* = (*F*_o_^2^ + 2*F*_c_^2^)/3
*S* = 1.03	(Δ/σ)_max_ < 0.001
3254 reflections	Δρ_max_ = 0.13 e Å^−^^3^
407 parameters	Δρ_min_ = −0.11 e Å^−^^3^
6 restraints	Extinction correction: *SHELXL97* (Sheldrick, 2008), Fc^*^=kFc[1+0.001xFc^2^λ^3^/sin(2θ)]^-1/4^
Primary atom site location: structure-invariant direct methods	Extinction coefficient: 0.015 (2)

### Special details

**Table:**

Geometry. All esds (except the esd in the dihedral angle between two l.s. planes) are estimated using the full covariance matrix. The cell esds are taken into account individually in the estimation of esds in distances, angles and torsion angles; correlations between esds in cell parameters are only used when they are defined by crystal symmetry. An approximate (isotropic) treatment of cell esds is used for estimating esds involving l.s. planes.
Refinement. Refinement of F^2^ against ALL reflections. The weighted R-factor wR and goodness of fit S are based on F^2^, conventional R-factors R are based on F, with F set to zero for negative F^2^. The threshold expression of F^2^ > 2sigma(F^2^) is used only for calculating R-factors(gt) etc. and is not relevant to the choice of reflections for refinement. R-factors based on F^2^ are statistically about twice as large as those based on F, and R- factors based on ALL data will be even larger.

### Fractional atomic coordinates and isotropic or equivalent isotropic displacement parameters (Å^2^)

**Table:**

	*x*	*y*	*z*	*U*_iso_*/*U*_eq_	
O1	0.4602 (3)	0.17321 (13)	0.29539 (14)	0.0654 (6)	
O2	0.0189 (3)	0.07939 (12)	0.68381 (15)	0.0651 (6)	
H2B	0.1181	0.0548	0.7126	0.098*	
O3	0.7089 (4)	0.48243 (11)	0.21582 (13)	0.0603 (5)	
O4	0.3140 (3)	0.69296 (12)	−0.13484 (15)	0.0649 (6)	
H4	0.4072	0.6910	−0.1747	0.097*	
O5	0.7330 (3)	0.31729 (12)	0.2971 (2)	0.0688 (6)	
N1	0.2364 (3)	0.27325 (12)	0.18320 (15)	0.0465 (5)	
N2	0.1558 (4)	0.24130 (13)	0.26688 (16)	0.0486 (5)	
N3	0.2760 (4)	0.48063 (14)	−0.21084 (17)	0.0611 (6)	
N4	0.3944 (4)	0.43505 (13)	0.32898 (16)	0.0540 (6)	
N5	0.3526 (4)	0.47696 (14)	0.24264 (17)	0.0565 (6)	
N6	0.2273 (4)	0.26319 (14)	0.73893 (17)	0.0574 (6)	
C1	0.2382 (5)	0.44061 (15)	−0.1264 (2)	0.0478 (6)	
C2	0.3964 (4)	0.39311 (15)	−0.0790 (2)	0.0500 (6)	
H2	0.5311	0.3887	−0.1047	0.060*	
C3	0.3556 (4)	0.35288 (15)	0.0050 (2)	0.0496 (6)	
H3	0.4637	0.3219	0.0349	0.059*	
C4	0.1567 (4)	0.35769 (13)	0.04580 (19)	0.0423 (6)	
C5	0.0014 (5)	0.40579 (16)	−0.0010 (2)	0.0523 (7)	
H5	−0.1325	0.4106	0.0253	0.063*	
C6	0.0391 (5)	0.44617 (15)	−0.0841 (2)	0.0540 (7)	
H6	−0.0688	0.4777	−0.1130	0.065*	
C7	0.4777 (6)	0.4754 (2)	−0.2563 (2)	0.0733 (9)	
H7A	0.5724	0.5152	−0.2327	0.110*	
H7B	0.4487	0.4785	−0.3281	0.110*	
H7C	0.5468	0.4291	−0.2386	0.110*	
C8	0.1119 (6)	0.5281 (2)	−0.2596 (3)	0.0777 (10)	
H8A	−0.0102	0.4989	−0.2867	0.117*	
H8B	0.1715	0.5544	−0.3131	0.117*	
H8C	0.0646	0.5627	−0.2118	0.117*	
C9	0.1034 (4)	0.31732 (14)	0.13410 (19)	0.0475 (6)	
H9	−0.0343	0.3238	0.1560	0.057*	
C10	0.2801 (4)	0.19211 (14)	0.32054 (19)	0.0446 (6)	
C11	0.1976 (4)	0.16137 (13)	0.41294 (18)	0.0403 (5)	
C12	0.3309 (4)	0.11208 (16)	0.4684 (2)	0.0518 (7)	
H12	0.4620	0.0979	0.4449	0.062*	
C13	0.2740 (5)	0.08319 (17)	0.5585 (2)	0.0537 (7)	
H13	0.3663	0.0501	0.5949	0.064*	
C14	0.0801 (5)	0.10396 (14)	0.59334 (19)	0.0486 (6)	
C15	−0.0585 (5)	0.15106 (16)	0.5375 (2)	0.0548 (7)	
H15	−0.1926	0.1634	0.5598	0.066*	
C16	0.0001 (4)	0.18011 (15)	0.4486 (2)	0.0510 (6)	
H16	−0.0937	0.2127	0.4120	0.061*	
C17	0.2277 (4)	0.30157 (14)	0.65040 (19)	0.0442 (6)	
C18	0.0467 (5)	0.34222 (16)	0.6109 (2)	0.0529 (7)	
H18	−0.0778	0.3437	0.6452	0.063*	
C19	0.0500 (5)	0.38022 (16)	0.5216 (2)	0.0565 (7)	
H19	−0.0734	0.4064	0.4965	0.068*	
C20	0.2329 (4)	0.38037 (14)	0.4682 (2)	0.0484 (6)	
C21	0.4131 (4)	0.34006 (14)	0.5066 (2)	0.0485 (6)	
H21	0.5370	0.3391	0.4718	0.058*	
C22	0.4125 (4)	0.30143 (15)	0.5953 (2)	0.0490 (6)	
H22	0.5359	0.2748	0.6193	0.059*	
C23	0.0321 (5)	0.25746 (19)	0.7900 (2)	0.0673 (9)	
H23A	−0.0182	0.3061	0.8051	0.101*	
H23B	0.0634	0.2302	0.8514	0.101*	
H23C	−0.0790	0.2323	0.7476	0.101*	
C24	0.4155 (6)	0.2222 (2)	0.7789 (2)	0.0712 (9)	
H24A	0.4520	0.1861	0.7304	0.107*	
H24B	0.3842	0.1976	0.8395	0.107*	
H24C	0.5364	0.2553	0.7933	0.107*	
C25	0.2250 (5)	0.42193 (15)	0.3751 (2)	0.0546 (7)	
H25	0.0908	0.4399	0.3476	0.065*	
C26	0.5169 (5)	0.50093 (14)	0.19151 (19)	0.0473 (6)	
C27	0.4562 (4)	0.55060 (13)	0.10541 (17)	0.0421 (6)	
C28	0.2589 (4)	0.58769 (15)	0.09028 (19)	0.0503 (6)	
H28	0.1542	0.5804	0.1351	0.060*	
C29	0.2144 (4)	0.63495 (16)	0.0107 (2)	0.0517 (7)	
H29	0.0806	0.6592	0.0021	0.062*	
C30	0.3686 (4)	0.64671 (14)	−0.05740 (18)	0.0456 (6)	
C31	0.5696 (4)	0.61128 (15)	−0.04239 (19)	0.0491 (6)	
H31	0.6753	0.6193	−0.0865	0.059*	
C32	0.6112 (4)	0.56435 (14)	0.03792 (19)	0.0462 (6)	
H32	0.7464	0.5411	0.0476	0.055*	
H2A	0.030 (3)	0.2603 (18)	0.284 (2)	0.080*	
H5C	0.213 (2)	0.4898 (19)	0.223 (2)	0.080*	
H5A	0.694 (5)	0.3625 (7)	0.297 (3)	0.080*	
H5B	0.617 (3)	0.2919 (14)	0.285 (3)	0.080*	

### Atomic displacement parameters (Å^2^)

**Table:**

	*U*^11^	*U*^22^	*U*^33^	*U*^12^	*U*^13^	*U*^23^
O1	0.0554 (12)	0.0931 (15)	0.0510 (11)	0.0225 (11)	0.0224 (9)	0.0161 (11)
O2	0.0711 (14)	0.0726 (14)	0.0561 (12)	−0.0083 (11)	0.0306 (11)	0.0069 (10)
O3	0.0715 (14)	0.0634 (12)	0.0472 (11)	0.0164 (11)	0.0119 (10)	0.0052 (9)
O4	0.0611 (13)	0.0811 (14)	0.0558 (12)	0.0156 (11)	0.0230 (10)	0.0279 (11)
O5	0.0549 (12)	0.0606 (12)	0.0941 (16)	0.0071 (10)	0.0228 (12)	−0.0077 (12)
N1	0.0485 (13)	0.0501 (11)	0.0426 (12)	−0.0022 (10)	0.0124 (10)	0.0019 (10)
N2	0.0461 (12)	0.0555 (12)	0.0461 (12)	0.0021 (11)	0.0149 (10)	0.0063 (10)
N3	0.0613 (16)	0.0686 (15)	0.0532 (15)	−0.0010 (12)	0.0045 (12)	0.0180 (13)
N4	0.0742 (17)	0.0493 (12)	0.0392 (12)	−0.0003 (11)	0.0093 (11)	0.0065 (10)
N5	0.0670 (15)	0.0566 (13)	0.0470 (13)	0.0007 (12)	0.0109 (12)	0.0151 (11)
N6	0.0596 (15)	0.0673 (15)	0.0471 (13)	−0.0063 (12)	0.0153 (11)	0.0144 (12)
C1	0.0507 (16)	0.0488 (14)	0.0429 (14)	−0.0061 (12)	−0.0004 (12)	−0.0005 (12)
C2	0.0466 (15)	0.0570 (15)	0.0470 (15)	−0.0005 (13)	0.0070 (12)	0.0040 (13)
C3	0.0486 (15)	0.0507 (14)	0.0495 (15)	0.0032 (12)	0.0052 (13)	0.0044 (12)
C4	0.0421 (14)	0.0442 (13)	0.0407 (13)	0.0021 (11)	0.0041 (11)	0.0012 (11)
C5	0.0446 (15)	0.0592 (16)	0.0539 (16)	0.0081 (12)	0.0076 (12)	0.0011 (13)
C6	0.0537 (17)	0.0557 (16)	0.0520 (16)	0.0112 (13)	0.0017 (14)	0.0067 (13)
C7	0.081 (2)	0.082 (2)	0.0582 (19)	−0.0092 (19)	0.0156 (17)	0.0109 (17)
C8	0.083 (2)	0.080 (2)	0.068 (2)	0.0059 (19)	−0.0020 (18)	0.0280 (18)
C9	0.0431 (14)	0.0523 (14)	0.0479 (15)	0.0009 (12)	0.0084 (12)	−0.0007 (12)
C10	0.0447 (15)	0.0486 (14)	0.0416 (14)	−0.0001 (11)	0.0102 (11)	−0.0040 (11)
C11	0.0405 (13)	0.0415 (12)	0.0398 (13)	0.0002 (10)	0.0073 (11)	−0.0042 (10)
C12	0.0474 (15)	0.0624 (16)	0.0477 (15)	0.0042 (13)	0.0155 (12)	−0.0019 (13)
C13	0.0568 (17)	0.0601 (16)	0.0460 (14)	0.0065 (13)	0.0135 (13)	0.0075 (13)
C14	0.0553 (16)	0.0485 (14)	0.0440 (14)	−0.0101 (13)	0.0154 (12)	−0.0038 (12)
C15	0.0459 (15)	0.0631 (17)	0.0582 (17)	−0.0015 (14)	0.0196 (13)	−0.0019 (14)
C16	0.0466 (15)	0.0539 (15)	0.0540 (15)	−0.0003 (12)	0.0116 (12)	−0.0015 (13)
C17	0.0456 (14)	0.0443 (12)	0.0429 (14)	−0.0077 (11)	0.0054 (11)	0.0036 (11)
C18	0.0466 (16)	0.0582 (16)	0.0561 (16)	−0.0023 (13)	0.0165 (13)	0.0041 (14)
C19	0.0514 (17)	0.0561 (16)	0.0626 (18)	0.0050 (13)	0.0087 (14)	0.0123 (14)
C20	0.0573 (17)	0.0447 (13)	0.0442 (15)	−0.0021 (12)	0.0103 (13)	0.0045 (12)
C21	0.0513 (16)	0.0502 (14)	0.0463 (14)	−0.0049 (12)	0.0165 (12)	0.0013 (12)
C22	0.0466 (15)	0.0527 (14)	0.0485 (15)	0.0020 (12)	0.0095 (12)	0.0061 (12)
C23	0.075 (2)	0.075 (2)	0.0553 (17)	−0.0181 (17)	0.0228 (16)	0.0084 (16)
C24	0.086 (2)	0.074 (2)	0.0537 (18)	0.0081 (18)	0.0090 (17)	0.0218 (16)
C25	0.0645 (19)	0.0485 (15)	0.0511 (17)	0.0020 (13)	0.0079 (14)	0.0081 (13)
C26	0.0670 (19)	0.0406 (13)	0.0351 (13)	0.0054 (12)	0.0090 (13)	−0.0043 (11)
C27	0.0557 (15)	0.0371 (12)	0.0349 (12)	0.0009 (11)	0.0109 (11)	−0.0040 (10)
C28	0.0543 (16)	0.0564 (15)	0.0431 (14)	−0.0005 (13)	0.0198 (12)	0.0027 (12)
C29	0.0468 (15)	0.0615 (16)	0.0490 (16)	0.0078 (12)	0.0165 (12)	0.0070 (13)
C30	0.0527 (15)	0.0495 (14)	0.0368 (13)	0.0015 (12)	0.0158 (11)	0.0041 (11)
C31	0.0488 (15)	0.0559 (15)	0.0453 (15)	0.0045 (12)	0.0191 (12)	0.0020 (12)
C32	0.0505 (16)	0.0465 (14)	0.0428 (14)	0.0080 (11)	0.0103 (12)	0.0010 (11)

### Geometric parameters (Å, °)

**Table:**

O1—C10	1.236 (3)	C10—C11	1.487 (3)
O2—C14	1.373 (3)	C11—C12	1.379 (4)
O2—H2B	0.8200	C11—C16	1.388 (3)
O3—C26	1.240 (3)	C12—C13	1.387 (4)
O4—C30	1.348 (3)	C12—H12	0.9300
O4—H4	0.8200	C13—C14	1.374 (4)
O5—H5A	0.852 (10)	C13—H13	0.9300
O5—H5B	0.853 (10)	C14—C15	1.374 (4)
N1—C9	1.278 (3)	C15—C16	1.378 (4)
N1—N2	1.391 (3)	C15—H15	0.9300
N2—C10	1.337 (3)	C16—H16	0.9300
N2—H2A	0.896 (10)	C17—C18	1.396 (4)
N3—C1	1.379 (3)	C17—C22	1.412 (3)
N3—C8	1.434 (4)	C18—C19	1.379 (4)
N3—C7	1.436 (4)	C18—H18	0.9300
N4—C25	1.283 (4)	C19—C20	1.389 (4)
N4—N5	1.384 (3)	C19—H19	0.9300
N5—C26	1.345 (3)	C20—C21	1.384 (4)
N5—H5C	0.903 (10)	C20—C25	1.449 (4)
N6—C17	1.371 (3)	C21—C22	1.375 (4)
N6—C24	1.435 (4)	C21—H21	0.9300
N6—C23	1.441 (3)	C22—H22	0.9300
C1—C6	1.400 (4)	C23—H23A	0.9600
C1—C2	1.403 (4)	C23—H23B	0.9600
C2—C3	1.380 (4)	C23—H23C	0.9600
C2—H2	0.9300	C24—H24A	0.9600
C3—C4	1.389 (4)	C24—H24B	0.9600
C3—H3	0.9300	C24—H24C	0.9600
C4—C5	1.395 (4)	C25—H25	0.9300
C4—C9	1.451 (4)	C26—C27	1.479 (4)
C5—C6	1.368 (4)	C27—C28	1.385 (4)
C5—H5	0.9300	C27—C32	1.396 (3)
C6—H6	0.9300	C28—C29	1.372 (4)
C7—H7A	0.9600	C28—H28	0.9300
C7—H7B	0.9600	C29—C30	1.391 (3)
C7—H7C	0.9600	C29—H29	0.9300
C8—H8A	0.9600	C30—C31	1.390 (4)
C8—H8B	0.9600	C31—C32	1.373 (4)
C8—H8C	0.9600	C31—H31	0.9300
C9—H9	0.9300	C32—H32	0.9300
			
C14—O2—H2B	109.5	O2—C14—C15	118.1 (2)
C30—O4—H4	109.5	C13—C14—C15	120.0 (2)
H5A—O5—H5B	107 (2)	C14—C15—C16	120.3 (3)
C9—N1—N2	114.3 (2)	C14—C15—H15	119.9
C10—N2—N1	118.5 (2)	C16—C15—H15	119.9
C10—N2—H2A	126 (2)	C15—C16—C11	120.8 (3)
N1—N2—H2A	115 (2)	C15—C16—H16	119.6
C1—N3—C8	121.3 (3)	C11—C16—H16	119.6
C1—N3—C7	122.1 (3)	N6—C17—C18	121.7 (2)
C8—N3—C7	116.5 (3)	N6—C17—C22	121.1 (2)
C25—N4—N5	114.0 (2)	C18—C17—C22	117.2 (2)
C26—N5—N4	120.7 (2)	C19—C18—C17	120.8 (2)
C26—N5—H5C	120 (2)	C19—C18—H18	119.6
N4—N5—H5C	119 (2)	C17—C18—H18	119.6
C17—N6—C24	121.0 (2)	C18—C19—C20	121.7 (3)
C17—N6—C23	121.0 (2)	C18—C19—H19	119.2
C24—N6—C23	117.8 (2)	C20—C19—H19	119.2
N3—C1—C6	121.0 (3)	C21—C20—C19	117.9 (2)
N3—C1—C2	121.8 (3)	C21—C20—C25	123.5 (2)
C6—C1—C2	117.3 (2)	C19—C20—C25	118.6 (3)
C3—C2—C1	121.2 (3)	C22—C21—C20	121.3 (2)
C3—C2—H2	119.4	C22—C21—H21	119.3
C1—C2—H2	119.4	C20—C21—H21	119.3
C2—C3—C4	121.4 (2)	C21—C22—C17	121.1 (2)
C2—C3—H3	119.3	C21—C22—H22	119.5
C4—C3—H3	119.3	C17—C22—H22	119.5
C3—C4—C5	117.0 (2)	N6—C23—H23A	109.5
C3—C4—C9	124.3 (2)	N6—C23—H23B	109.5
C5—C4—C9	118.7 (2)	H23A—C23—H23B	109.5
C6—C5—C4	122.4 (3)	N6—C23—H23C	109.5
C6—C5—H5	118.8	H23A—C23—H23C	109.5
C4—C5—H5	118.8	H23B—C23—H23C	109.5
C5—C6—C1	120.8 (3)	N6—C24—H24A	109.5
C5—C6—H6	119.6	N6—C24—H24B	109.5
C1—C6—H6	119.6	H24A—C24—H24B	109.5
N3—C7—H7A	109.5	N6—C24—H24C	109.5
N3—C7—H7B	109.5	H24A—C24—H24C	109.5
H7A—C7—H7B	109.5	H24B—C24—H24C	109.5
N3—C7—H7C	109.5	N4—C25—C20	123.1 (3)
H7A—C7—H7C	109.5	N4—C25—H25	118.5
H7B—C7—H7C	109.5	C20—C25—H25	118.5
N3—C8—H8A	109.5	O3—C26—N5	121.5 (2)
N3—C8—H8B	109.5	O3—C26—C27	122.1 (2)
H8A—C8—H8B	109.5	N5—C26—C27	116.4 (2)
N3—C8—H8C	109.5	C28—C27—C32	117.6 (2)
H8A—C8—H8C	109.5	C28—C27—C26	124.1 (2)
H8B—C8—H8C	109.5	C32—C27—C26	118.2 (2)
N1—C9—C4	123.2 (2)	C29—C28—C27	121.5 (2)
N1—C9—H9	118.4	C29—C28—H28	119.2
C4—C9—H9	118.4	C27—C28—H28	119.2
O1—C10—N2	121.4 (2)	C28—C29—C30	120.3 (2)
O1—C10—C11	120.5 (2)	C28—C29—H29	119.9
N2—C10—C11	118.1 (2)	C30—C29—H29	119.9
C12—C11—C16	117.9 (2)	O4—C30—C31	123.4 (2)
C12—C11—C10	117.1 (2)	O4—C30—C29	117.5 (2)
C16—C11—C10	124.9 (2)	C31—C30—C29	119.2 (2)
C11—C12—C13	121.6 (3)	C32—C31—C30	119.7 (2)
C11—C12—H12	119.2	C32—C31—H31	120.2
C13—C12—H12	119.2	C30—C31—H31	120.2
C14—C13—C12	119.3 (3)	C31—C32—C27	121.8 (2)
C14—C13—H13	120.3	C31—C32—H32	119.1
C12—C13—H13	120.3	C27—C32—H32	119.1
O2—C14—C13	122.0 (3)		

### Hydrogen-bond geometry (Å, °)

**Table:**

*D*—H···*A*	*D*—H	H···*A*	*D*···*A*	*D*—H···*A*
N5—H5C···O2^i^	0.90 (1)	2.56 (3)	3.171 (3)	125 (3)
N2—H2A···O5^ii^	0.90 (1)	2.12 (1)	3.003 (3)	168 (3)
O4—H4···O1^iii^	0.82	1.90	2.688 (2)	160
O2—H2B···O3^iv^	0.82	1.89	2.693 (3)	166
O5—H5A···O3	0.85 (1)	2.43 (2)	3.178 (3)	146 (3)
O5—H5A···N4	0.85 (1)	2.34 (2)	3.038 (3)	140 (3)
O5—H5B···O1	0.85 (1)	2.36 (2)	3.100 (3)	145 (3)
O5—H5B···N1	0.85 (1)	2.61 (2)	3.375 (3)	150 (3)

Symmetry codes: (i) −*x*, *y*+1/2, −*z*+1; (ii) *x*−1, *y*, *z*; (iii) −*x*+1, *y*+1/2, −*z*; (iv) −*x*+1, *y*−1/2, −*z*+1.
